# Burnout levels and correlates among healthcare providers in Syria following the 2023 Turkish–Syrian earthquakes: A cross‐sectional study

**DOI:** 10.1002/hsr2.2080

**Published:** 2024-04-29

**Authors:** Caroline Almohsen, Jameel Soqia, Samer Mohsen

**Affiliations:** ^1^ Department of Clinical Psychology, Faculty of Health Sciences Damascus University Damascus Syria; ^2^ Faculty of Medicine Damascus University Damascus Syria; ^3^ Department of Audiology, Faculty of Health Sciences Damascus University Damascus Syria

**Keywords:** burnout, earthquake, mental health, Syria, war

## Abstract

**Background and Aims:**

This article examines the prevalence of burnout among healthcare providers in the aftermath of the recent earthquakes in Syria and Turkey and explores the associated risk factors.

**Methods:**

This cross‐sectional study included 270 healthcare providers in three Syrian cities damaged by earthquakes. Participants were asked to fill out a validated questionnaire on the fifth day of emergency response using the Geldard Occupational Burnout questionnaire.

**Results:**

The mean score for the Geldard Occupational Burnout Questionnaire was 129.79, with 81.4% indicating moderate burnout risk and only 3% indicating high risk. Gender was not significantly associated with burnout, but there was a significant difference in burnout scores between city groups, with Latakia scoring significantly lower than Aleppo.

**Conclusion:**

This study highlights the prevalence of burnout among healthcare providers in the aftermath of an earthquake in Syria, with the majority having a moderate risk of burnout. Gender was not significantly associated with burnout risk. Further research is needed to develop effective interventions and address study limitations. The study emphasizes the importance of prioritizing healthcare providers' mental health to ensure high‐quality care after natural disasters.

## 1 INTRODUCTION

Natural disasters such as earthquakes have the potential to cause widespread devastation, leaving many communities in need of urgent medical care.^1^, ^2^, ^3^, ^4^ In the aftermath of such events, healthcare providers play a crucial role in responding to the needs of those affected. However, the demands placed on healthcare providers during disaster response can lead to high levels of stress, which can increase the risk of burnout.^5^, ^6^, ^7^ Burnout is a state of being characterized by decreased motivation, negative attitudes towards oneself and others, and physical, emotional, or mental exhaustion. It occurs as a result of prolonged and intense physical or mental exertion, or an excessive workload, which eventually leads to stress and tension taking their toll on an individual.^6^, ^7^ Burnout can have serious consequences for healthcare providers' well‐being, including the increased risk of mental health problems and decreased job satisfaction.^6^, ^7^

According to various studies, health workers have been found to experience the highest rates of burnout compared to other professionals, with prevalence ranging from 30% to 70%.^5^ Other studies reported a high prevalence of burnout levels among physicians (25.97%) in Italy after 6 years of the 2009 L'Aquila earthquake^7^; 55% in the workers in essential services after the Hurricanes Irma and Maria,^8^ and 15.9% among public servants after the great east Japan earthquake in 2011.^9^

Syria and Turkey were struck by a 7.8 magnitude earthquake in the early hours of February 6, 2023 at the city of Gaziantep, followed hours later by 7.5 magnitude earthquake in the Elbistan district, which directly impacted more than 26 million people across Syria and Turkey, and many more individuals indirectly.^10^ Thousands of peoples' homes have been destroyed in Syria and Turkey, while roads, hospitals and other facilities have been badly damaged, even surrounding countries like Iraq, Jordan, Lebanon, and Cyprus have been significantly impacted.^10^ Over 35,000 individuals have been reported dead in Turkey and more than 5000 in Syria is expected to have died, it is believed that the real numbers are much higher as there is no official statistics in Syria.^10^, ^11^ Amidst the current situation, 3000 people have found respite in temporary shelter, while a staggering 380,000 others have sought refuge in schools and education facilities.^10^, ^11^

Understanding the impact of earthquakes on healthcare providers' burnout is crucial for developing effective support strategies for their mental health and ensuring the delivery of high‐quality care. Previous studies have shown that disaster responders are at high risk of developing burnout, which is a syndrome of emotional exhaustion, depersonalization, and reduced personal accomplishment that can negatively affect their mental and physical health, as well as their professional performance and quality of care.^7^, ^8^, ^9^ This study examines the prevalence of burnout among healthcare providers 5 days post‐earthquake, exploring associated risk factors and discussing potential interventions. While previous studies, such as those by Alzailai et al.^12^ and Kawashima et al.^13^ have documented the high risk of burnout in disaster responders, they primarily focus on long‐term effects due to chronic stressors or repeated traumatic events like war, terrorism, or epidemics. Our research contributes to the existing literature by assessing the acute and immediate impact of a single, large‐scale disaster on healthcare providers' burnout risk. This baseline assessment enables longitudinal research to track burnout over time among this population, offering insights into temporal patterns and variations postdisaster.

## 2 METHODS

### 2.1 Study design, participants, and sampling

This cross‐sectional study included healthcare providers in three Syrian cities that have been damaged directly; Aleppo, Hama, and Latakia. During this study, a total of 270 healthcare providers were available in these sites. They were asked to fill the questionnaire at the fifth day (10/2/2023) of emergency quick response after Syrian–Turkish earthquakes. We selected healthcare providers from three primary emergency response sites in Aleppo, Lattakia, and Hama for this study based on several criteria. These sites were chosen due to their strategic importance in the earthquake relief efforts, and their accessibility for the research team. We aimed to ensure that our sample represented a wide range of healthcare professionals involved in different aspects of the disaster response. All eligible healthcare providers, defined as those above 18 years old and who had worked continuously for 5 days following the earthquake, were invited to participate. Out of 270 eligible providers, 237 agreed to participate, resulting in an 87.7% response rate. This high response rate suggests that our findings may be reflective of the broader population of healthcare providers involved in the earthquake response. Healthcare providers at these sites had medical backgrounds, such as physicians, nurses, or paramedics. Any individual who was over the age of 18, among healthcare providers at earthquakes' emergency response sites, and worked continually for 5 days there was eligible for this study, where participation in the survey was voluntary. This study was designed with the Strengthening the Reporting of Observational Studies in Epidemiology (STROBE) guidelines. This study was designed with the Strengthening the Reporting of Observational Studies in Epidemiology (STROBE) guidelines.

### 2.2 Data collection

Data was collected using a validated‐structured questionnaire presented by papers to participants. Participants' verbal and written consent was obtained with ensuring to maintain the secrecy of information, 4–7 min was the average time needed to complete the questionnaire.

### 2.3 Questionnaire

A validated questionnaire was adopted from surveys in similar previous studies^14^ as a point of reference for validation regarding Syrian community. The questionnaire consisted of two sections:

Section 1: Included demographic characteristics (Gender and city).

Section 2: This section assessed participants' Burnout status using The Geldard occupational burnout questionnaire, a widely used tool in current research, comprised the second section. The questionnaire consists of 40 items, and aims to assess the level of occupational burnout experienced by employees.^14^ The Likert scale is used to score each item on a scale of 1–7, with 1 indicating “absolutely agree” and 7 indicating “absolutely disagree.” The resulting scores, ranging from 40 to 280 points, provide an indication of the level of occupational burnout experienced by the individual, with lower scores suggesting lower levels of burnout and higher scores suggesting the opposite. The Geldard Occupational Burnout Questionnaire scores individuals based on their responses to 40 items, each scored on a Likert scale of 1–7. Scores between 40 and 109 indicate low risk of occupational burnout, scores between 110 and 165 indicate moderate risk, and scores between 166 and 280 indicate high risk.^15^, ^16^ The reliability and validity of this questionnaire have been evaluated in multiple previous studies.^14^, ^15^, ^16^

To ensure precision in meaning and concepts, the survey was adopted and validated for the Arabic language. It was initially translated into Arabic, the local language, by language experts and then re‐translated back into English, five experts have approved the face validity of the translated version. A pilot study was conducted with 57 healthcare providers at Damascus University Hospital which had not suffered from direct impact of the earthquake, and the questionnaire was subsequently revised based on primary statistical analyses. Furthermore, to assess the reliability of the survey, Cronbach's Alpha test was employed, yielding a good reliability of 0.812. The majority of the survey questions were presented in a closed format.

### 2.4 Ethical approval details and informed consent

Our research project was conducted on human subjects in compliance with the ethical standards outlined in the Declaration of Helsinki. The Ethical Committee at Damascus University's Faculty of Health Sciences granted approval for the study (ID:2541/MBD), and all participants were fully informed and provided written consent to take part. Participation was voluntary and anonymous, and the survey's objectives and questions were explained to all participants. To ensure privacy and confidentiality, no personal data was collected during the study.

### 2.5 Statistical analysis

We used SPSS version 25 for data analysis. Demographics characteristics were described using frequency and percentage for categorical variables. Independent *t*‐test and one‐way analysis of variance (ANOVA) were used to compare means of continuous variables. A *p*‐value of less than 0.05 was considered statistically significant. Partial eta squared was used to determine the effect size of the observed differences in mean scores. We reported partial eta squared values along with *F*‐statistics and *p*‐values for each factor in our ANOVA results. We also interpreted the magnitude of partial eta squared values based on Cohen's guidelines,^17^ which suggest that values of 0.01, 0.06, and 0.14 represent small, medium, and large effects, respectively.

## 3 RESULTS

Data was gathered from 237 individuals (*N* = 237), with females accounting for 55% of the sample (*n* = 130) and males accounting for 45% (*n* = 107). The majority of participants (67.6%, *n* = 160) worked in Latakia, while a smaller number worked in Hama (16.4%, *n* = 39) and Aleppo (16%, *n* = 38).

**Figure 1:**
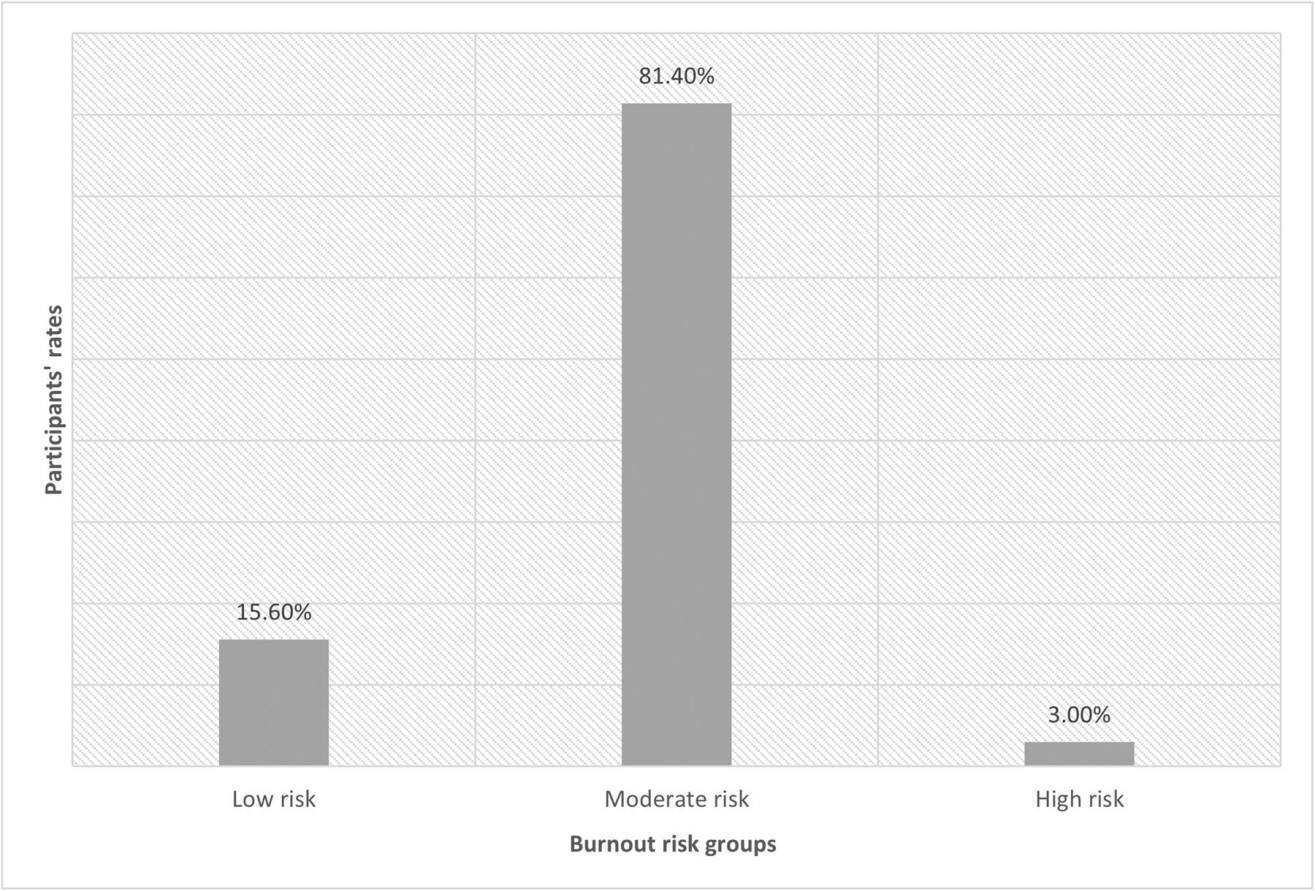
Participants' Burnout risk divided into three groups based on Geldard Occupational Burnout questionnaire scores.

The mean score for the Geldard Occupational Burnout Questionnaire was 129.79 (SD = 21.89). Of the sample, 15.6% (*n* = 37) had scores between 40 and 109, indicating a low risk of occupational burnout, while 81.4% (*n* = 193) had scores between 110 and 165, indicating a moderate risk. Only 3% (*n* = 7) had scores between 166 and 280, indicating a high risk of burnout (Figure 1).

Gender was not significantly associated with burnout risk, as there was no significant difference in scores between males and females (*t*(235) = −0.133, *p* = 0.894). A one‐way ANOVA was conducted to compare the difference in burnout scores between city groups, which revealed a significant difference between groups (*F*(2,234) = 6.260, *p* < 0.001, partial eta squared = 0.05) (Table 1). However, it is important to note that the effect size (as indicated by partial eta squared) is relatively moderate, suggesting that city groups explain a moderate portion of the variance in burnout scores (Table 1). A Tukey post hoc test indicated that burnout scores were significantly lower in the Latakia group (*M* = 126.45 ± 20.429, *p* < 0.001) compared to the Aleppo group (*M* = 138.21 ± 14.38).

**Table 1:** Mean burnout scores and standard deviations (SDs) for three groups of Syrian health workers based on their location.

	Lattakia group	Aleppo group	Hama group	*p*‐Value (One‐way ANOVA)	*F*‐statistics	Effect size (partial eta squared)
Burnout scores (SDs)	126.45 (20.42)	138.21 (14.38)	135.53 (29.83)	<0.001	6.26	0.05

Abbreviation: ANOVA, analysis of variance.

## 4 DISCUSSION

The aim of this study was to examine the prevalence of burnout among healthcare providers in the aftermath of an earthquake, and identify risk factors associated with burnout in this context. The results of this study have significant implications for understanding the impact of natural disasters on healthcare providers and for developing strategies to support their mental health and ensure the provision of high‐quality care.^6^, ^18^

The findings of this study reveal that the majority of healthcare providers in the sample (81.4%) had a moderate risk of burnout, while a small proportion had a low (15.6%) or high risk (3%) of burnout. This indicates that the aftermath of an earthquake can have a significant impact on the mental health of healthcare providers and underscores the importance of addressing this issue to ensure the provision of high‐quality care. The results of our study align with previous research that has found a high prevalence of burnout among healthcare providers,^6^, ^7^, ^19^, ^20^ public servants,^9^ and workers in essential services following natural disasters.^8^ It is worth noting that direct comparisons between studies are challenging due to differences in the scales used to measure burnout. Notably, there is a significant gap in burnout research in Syria, with only one study from 2019 exploring burnout among resident physicians and finding a high prevalence of burnout.^21^

Interestingly, gender was not significantly associated with burnout risk in this study. This finding suggests that both male and female healthcare providers are vulnerable to burnout in the aftermath of an earthquake, highlighting the need for gender‐sensitive approaches to supporting the mental health of healthcare providers. This finding is different from a study conducted in Pakistan by Ehring et al.,^22^ which women found to show higher levels of Burnout.

An intriguing aspect of our findings was the variance in burnout scores among healthcare providers from different cities. Providers in Latakia reported significantly lower burnout scores compared to their counterparts in Aleppo. While this discrepancy could partly be attributed to the differential impact of the earthquake on these cities—with Aleppo experiencing more severe effects.^10^, ^23^ it is also necessary to consider other contributing factors. These may include, but are not limited to, pre‐existing infrastructure, availability of resources, and the level of social support systems in place.^23^ Such factors could influence the mental health outcomes of healthcare providers and highlight the need for interventions tailored to the unique challenges faced in each location.

### 4.1 Limitation

Our study provides insights into the prevalence of burnout among healthcare providers in the aftermath of an earthquake. However, it is imperative to note several limitations that future research should address. We did not account for the subjects' prior traumatic experiences, personal circumstances such as marital, work, and financial status, or the loss of family members—all of which could significantly affect mental health outcomes. The sensitive and insecure context of the study, set in a region with ongoing conflict and violence, posed ethical and practical challenges in collecting comprehensive personal data. This limitation restricts the generalizability and comparability of our findings. Moreover, the absence of a control group before the earthquake underscores the necessity for comparative studies to discern the disaster's effects more clearly. Lastly, our reliance on self‐reported questionnaires introduces potential variance, warranting cautious interpretation of the results.^24^

Our study did not account for prior traumatic experiences, personal circumstances, or loss of family members, which can impact mental health outcomes. Additionally, conducting research in conflict‐affected regions posed ethical and practical challenges, affecting data collection. Furthermore, the absence of a pre‐earthquake control group limits our ability to fully discern the disaster's effects. Lastly, our reliance on self‐reported questionnaires introduces potential variance, warranting cautious interpretation of the results.

### 4.2 Recommendation

To effectively support the mental health of healthcare providers during the early stages of natural disasters, it is imperative to develop tailored interventions that are initiated promptly. These interventions must consider gender‐sensitive approaches and prioritize immediate needs. Research should focus on identifying contextual factors that influence burnout risk specifically during the acute phase of disasters. Future studies ought to incorporate a broader range of variables and employ a variety of methods to assess burnout accurately. Moreover, conducting comparative studies with control samples early in the disaster timeline will provide a clearer understanding of the initial effects on healthcare providers' burnout risk.

## 5 CONCLUSION

The aftermath of natural disasters can have a significant impact on the mental health of healthcare providers. This study sheds light on the prevalence of burnout among healthcare providers in the aftermath of an earthquake in Syria, revealing that the majority of healthcare providers in the sample had a moderate risk of burnout. Gender was not found to be significantly associated with burnout risk, and healthcare providers working in different cities had different burnout scores, highlighting the importance of tailoring interventions to the specific needs of healthcare providers in different locations. While this study provides valuable insights into the impact of natural disasters on healthcare providers, further research is needed to develop targeted interventions and address the limitations of the study. Overall, these findings underscore the need to prioritize the mental health of healthcare providers to ensure the provision of high‐quality care in the aftermath of natural disasters.

## AUTHOR CONTRIBUTIONS

**Caroline Almohsen**: Investigation; conceptualization; methodology; resources; visualization; data curation. **Jameel Soqia**: Investigation; writing—original draft; writing—review & editing; validation; formal analysis; software; data curation. **Samer Mohsen**: Supervision; writing—review & editing; validation; methodology; investigation; conceptualization; project administration.

## CONFLICT OF INTEREST STATEMENT

The authors declare no conflict of interest.

## TRANSPARENCY STATEMENT

The lead author Jameel Soqia affirms that this manuscript is an honest, accurate, and transparent account of the study being reported; that no important aspects of the study have been omitted; and that any discrepancies from the study as planned (and, if relevant, registered) have been explained.

## Data Availability

The data that support the findings of this study are available on request from the corresponding author. The data are not publicly available due to privacy or ethical restrictions. The data sets generated and analyzed during the current study are not publicly available to protect participants' privacy but are available from the corresponding author on reasonable request.
